# Cohort Multiple Randomised Controlled Trials (cmRCT) design: efficient but biased? A simulation study to evaluate the feasibility of the Cluster cmRCT design

**DOI:** 10.1186/s12874-016-0208-1

**Published:** 2016-08-26

**Authors:** Alexander Pate, Jane Candlish, Matthew Sperrin, Tjeerd Pieter Van Staa, on behalf of GetReal Work Package 2

**Affiliations:** 1 Health eResearch Centre, Farr Institute for Health Informatics Research, University of Manchester, 1.003 Vaughan House, Manchester, M13 9PL, UK; 2 Utrecht Institute of Pharmaceutical Sciences, Utrecht University, Utrecht, The Netherlands

**Keywords:** Trials within Cohorts, Cohort multiple randomised controlled trial, Cluster, Pragmatic, Instrumental variable

## Abstract

**Background:**

The Cohort Multiple Randomised Controlled Trial (cmRCT) is a newly proposed pragmatic trial design; recently several cmRCT have been initiated. This study tests the unresolved question of whether differential refusal in the intervention arm leads to bias or loss of statistical power and how to deal with this.

**Methods:**

We conduct simulations evaluating a hypothetical cluster cmRCT in patients at risk of cardiovascular disease (CVD). To deal with refusal, we compare the analysis methods intention to treat (ITT), per protocol (PP) and two instrumental variable (IV) methods: two stage predictor substitution (2SPS) and two stage residual inclusion (2SRI) with respect to their bias and power. We vary the correlation between treatment refusal probability and the probability of experiencing the outcome to create different scenarios.

**Results:**

We found ITT to be biased in all scenarios, PP the most biased when correlation is strong and 2SRI the least biased on average. Trials suffer a drop in power unless the refusal rate is factored into the power calculation.

**Conclusions:**

The ITT effect in routine practice is likely to lie somewhere between the ITT and IV estimates from the trial which differ significantly depending on refusal rates. More research is needed on how refusal rates of experimental interventions correlate with refusal rates in routine practice to help answer the question of which analysis more relevant. We also recommend updating the required sample size during the trial as more information about the refusal rate is gained.

## Background

Randomised controlled trials (RCTs) often fail to meet recruitment targets and are costly [1]. This problem can be even more prevalent in comparative effectiveness research where more patients are needed to detect smaller differences between treatments. Furthermore, the results from most randomised controlled trials may not be generalisable to routine practice [2], yet we use the results from these trials to inform clinical decision making [3]. There is a clear need for more pragmatic trials which are cost efficient, integrated with routine clinical care, have less stringent entry criteria and can address the clinical questions that current RCTs cannot [4–7].

The Cohort Multiple Randomised Controlled Trial (cmRCT) design can simplify the recruitment and conduct of trials compared with current RCTs. It was first proposed in 2010 [8], and is beginning to be used in practice with a total of 5 registered trials from 7 cohorts [9–16]. In this design, a large cohort is identified (e.g., patients at high risk of cardiovascular disease [CVD]) and followed using routinely collected data such as electronic health records [17]. The same cohort can be used for multiple interventions. Each intervention is offered to a randomly selected sample of patients eligible for that intervention, who are then compared with the rest of the eligible patients from the cohort that are still being treated as usual [8]. Randomisation can occur either at a patient or a cluster (site) level. The cluster design can offer dramatically improved accrual [1] and can further reduce costs through the implementation of the interventions in fewer places; cluster designs are the focus of this paper.

The main advantages of cmRCTs are the low cost of recruiting the control group, the possibility to use it for multiple trials and the comparison of interventions to real life practice (the control group are not contacted for further consent). However, refusal to participate in the trial happens post randomisation so excluding these patients may result in selection bias. Alternatively an intention to treat (ITT) analysis could be used; however the refusal rates in a cmRCT may not reflect those in routine practice as the intervention may be viewed as experimental. In this case the ITT effect may lack interpretation outside the trial setting. Depending on the refusal rate it may be preferable to calculate the effect of accepting the treatment, this will be referred to as the treatment effect for the remainder of this paper. Refusal can cause a loss of statistical power and a bias in the estimation of the treatment effect, particularly if it is correlated with the outcome of interest. Instrumental variable analysis (IV) is a method to account for unmeasured confounding in epidemiological studies [18, 19] and can for non-compliance in RCTs [20, 21]. It is also applicable to the problem of treatment refusal in a cmRCT setting. The aim of this paper is (1) to estimate the extent of bias and loss of statistical power with various refusals scenarios, (2) to test the robustness of IV methods to correct for bias due to refusals and (3) devise strategies to account for loss in power. There is currently very little literature on the topic of cmRCTs, we provide practical recommendations to trial designers and decision makers on the conditions under which cluster cmRCT is a viable design for point of care trials and which statistical analysis methods to use.

## Methods

A series of simulations are performed using Base SAS 9.4 Software in which cluster cmRCTs are conducted. In order to provide more realistic simulations they are based on an example of a cohort of patients at high risk of developing cardiovascular disease (CVD) and eligible for lipid lowering drugs according to the relevant criteria in the principal UK guidelines [22]. A novel intervention is tested against treatment as usual with a primary outcome of the time until a CVD event. This is an outcome that is of direct importance to a patient and may be identified with routinely collected data. Three patient characteristics are simulated: probability of refusing the intervention treatment, the risk of having a CVD event, and the time to death or censoring. Different scenarios are created by changing the average refusal probability of the population and changing the correlation between individuals’ risk of having an event and their probability of refusing treatment. The probability of a clinician refusing to offer the treatment to each patient is also simulated, and correlated to varying extents with patient risk. Once the patient characteristics have been generated, trial data is simulated through the same process of a cmRCT: treatment randomisation, refusal of treatment, application of intervention to those who accept and then the generation of times until an event. Weibull distributions are used to generate survival times. Each of the analysis methods explained in Analysis Methods are then applied to the simulated trial data to estimate the intervention effect. The exact simulation process is detailed in Simulation procedure.

### Analysis methods

Four different methods for the analysis of a cluster cmRCT are tested. The methods are ITT, per protocol (PP) and two IV methods. ITT is the recommended method of analysis in pragmatic trials [5, 23, 24] analysing the groups based on the random treatment allocation. PP defines the treatment groups on the basis of the actual treatment received, with only those who follow the allocated treatment included in the analysis. The two IV methods tested are the two stage predictor substitution (2SPS) and two stage residual inclusion (2SRI) as outlined practically by Terza et al., [25]. They are both two stage modelling techniques and start by fitting a first stage model with treatment allocation as the explanatory variable and treatment received as the dependent variable (here treatment allocation acts as the IV). This model is then used to calculate the predicted values for treatment received and the residuals. In 2SPS, a second stage model is fitted to the outcome data using the predicted values for treatment received as the explanatory variables. In 2SRI, the second stage model is fitted to the outcome data using both the residuals and the actual treatment received as explanatory variables. The standard errors of parameter estimates in two stage modelling procedures are too small hence non-parametric bootstrapping [26] should be used to calculate them. IV estimators were the chosen method to estimate the causal effect as IV methods are believed to perform well in RCTs with non-compliance with assumptions more easily argued to hold. [27]. There is a wealth of literature on the theoretical properties of causal effect estimates and IVs [18–21, 28] which is not recited in this paper. Instead the performances of the four different analysis methods in a variety of scenarios are evaluated with respect to bias, standard error and statistical power. We define bias as the error in the estimation of the treatment effect as defined in section 1 (effect of accepting treatment).

### Simulation procedure

Table 1 contains details of all variables used in the simulations. The cluster size chosen is *J* = 620 to match the average number of eligible patients per UK practice. This is calculated using published figures on GP practice size from the Health and Social Care Information Centre (HSCIC) [29] and statistics on the prevalence of CVD from the National Institute for Health and Care Excellence (NICE) [30]. The cluster size *J* is constant as it has been shown that variable cluster size has no effect on the results in terms of bias [31, 32]. The variances of the individual and cluster level random effects, σ_ε_^2^ and *σ*_*u*_^2^, and the shape and scale of the Weibull distribution for time to CVD event, *λ*_*c*_ and *γ*_*c*_, are chosen to match the mean 10-year CVD risk to published figures of 21.1 % (standard deviation 8.6 %) [22]. The mortality (censoring distribution) shape and scale, *γ*_*m*_ and *λ*_*m*_, and the variances σ_ε_^2^ and *σ*_*u*_^2^ give censoring of 5 % of all events and a correlation of 0.25 between *T*_*ik*_^*c*^ and *T*_*ik*_^*m*^ to represent informative censoring.

**Table 1:** Description of all variables used in simulation

Number of patients in cohort, control arm and intervention arm	N, N_con_, N_int_
Number of clusters in trial	K
Size of each cluster	J = 620
Treatment allocated to k^th^ cluster	Z_k_ = 0/1 for control/intervention
Treatment received by i^th^ individual from k^th^ cluster	X_ik_ = 0/1 for control/intervention
Time until CVD event for i^th^ individual from the k^th^ cluster	${\mathrm{T}}_{\mathrm{ik}}^{\mathrm{c}}\sim \mathrm{Weibull}\left({\upgamma}_{\mathrm{c}},{\uplambda}_{\mathrm{c}}{\mathrm{e}}^{-\left(\upbeta {\mathrm{X}}_{\mathrm{ik}}+{\upvarepsilon}_{\mathrm{ik}}+{\mathrm{U}}_{\mathrm{k}}\right)/{\upgamma}_{\mathrm{c}}}\right)$
Time until mortality (censoring distribution) for the i^th^ individual from the k^th^ cluster	${\mathrm{T}}_{\mathrm{ik}}^{\mathrm{m}}\sim \mathrm{Weibull}\left({\upgamma}_{\mathrm{m}},{\uplambda}_{\mathrm{m}}{\mathrm{e}}^{-\left({\upvarepsilon}_{\mathrm{ik}}+{\mathrm{U}}_{\mathrm{k}}\right)\kern0.1em /\kern0.1em {\upgamma}_{\mathrm{m}}}\right)$
Common baseline hazard function for time until CVD event	${\mathrm{h}}^{\mathrm{c}}\left(\mathrm{t}\right)={\upgamma}_{\mathrm{c}}{\mathrm{t}}^{\upgamma_{\mathrm{c}}-1}/\kern0.1em {\uplambda_{\mathrm{c}}}^{\upgamma_{\mathrm{c}}},\kern1em {\upgamma}_{\mathrm{c}}=1.2,{\uplambda}_{\mathrm{c}}=36$
Common baseline hazard function for time until mortality	${\mathrm{h}}^{\mathrm{m}}\left(\mathrm{t}\right)={\upgamma}_{\mathrm{m}}{\mathrm{t}}^{\upgamma_{\mathrm{m}}-1}/{\uplambda_{\mathrm{m}}}^{\upgamma_{\mathrm{m}}},\kern0.5em {\upgamma}_{\mathrm{m}}=1.2,{\uplambda}_{\mathrm{m}}=55$
Individual hazard function for time until CVD event	${\mathrm{h}}_{\mathrm{ik}}^{\mathrm{c}}\left(\mathrm{t}\right)={\mathrm{h}}^{\mathrm{c}}\left(\mathrm{t}\right){\mathrm{e}}^{\left({\upvarepsilon}_{\mathrm{ik}}+{\mathrm{U}}_{\mathrm{k}}+\upbeta {\mathrm{X}}_{\mathrm{ik}}\right)}$
Individual hazard function for time until mortality	${\mathrm{h}}_{\mathrm{ik}}^{\mathrm{m}}\left(\mathrm{t}\right)={\mathrm{h}}^{\mathrm{m}}\left(\mathrm{t}\right){\mathrm{e}}^{\left({\upvarepsilon}_{\mathrm{ik}}+{\mathrm{U}}_{\mathrm{k}}\right)}$
Individual level random effects	*ɛ*_*ik*_ ∼ *N*(0, *σ*_*ɛ*_^2^)
Cluster level random effects	*U*_*k*_ ∼ *N*(0, *σ*_*u*_^2^)
Intervention effect	*β* = − 0.32
Ten year risk of a CVD event	r_ik_ = P(T_ik_^c^ < 10| X_ik_ = 0, ε_ik_, U_k_)
Individual and average probability of patient refusing treatment	${p}_{ik},\;p={\displaystyle {\sum}_{i,k}\frac{p_{ik}}{N}}$
Individual and average probability of clinician refusing to offer treatment	${q}_{ik},\kern0.5em q={\displaystyle {\sum}_{i,k}\frac{q_{ik}}{N}}$
Correlation between patient refusal probability and patient risk	*ρ*_*p*_
Correlation between clinician refusal probability and patient risk	*ρ*_*q*_
Censoring indicator	*C*_*ik*_ = *I*(*T*_*ik*_^*c*^ ≥ *min*(*T*_*ik*_^*m*^, *T*_*max*_))
Trial follow up time	*T*_*max*_ = 3
Random variable observed for each patient	*Y*_*ik*_ = *min*(*T*_*ik*_^*c*^, *T*_*ik*_^*m*^, *T*_*max*_)

For each scenario detailed previously, the following procedure was implemented:

- For j = 1,2,…,1000:
  1. Generate the random effects *ɛ*_*ik*_ and *U*_*k*_ for each patient and cluster. i = 1,2,…,I. k = 1,2,…,K.
  2. For each patient, calculate unique 10 year risks (under the counterfactual scenario of receiving standard care) of a CVD event, *r*_*ik*_.
  3. Assign patient and clinician refusal probabilities *p*_*ik*_ and *q*_*ik*_
    - Order patients by their risk, *r*_*ik*_.
    - Assign refusal probabilities sequentially in a linear fashion between the lower limit (LL) and upper limit (UL) such that such that ∑ *p*_*ik*_/*N* = *p* and ∑ *q*_*ik*_/*N* = *q*.
  4. Randomise treatment allocation *Z*_*k*_ to control or intervention on a 4:1 basis. *Z*_*k*_ = 0/1 if assigned to control/intervention.
  5. Generate the treatment received where *X*_*ik*_ = 0/1 if control/intervention is received. If Z_k_ = 0 then X_ik_ = 0, if *Z*_*k*_ = 1 then *X*_*ik*_ = min {*Bernoulli*(1 − *p*_*ik*_), *Bernoulli*(1 − *q*_*ik*_)}.
  6. Apply intervention effect *β* and random effects to the hazard function, ${\mathrm{h}}_{\mathrm{ik}}^{\mathrm{c}}\left(\mathrm{t}\right)={\mathrm{h}}^{\mathrm{c}}\left(\mathrm{t}\right)\kern0.1em {\mathrm{e}}^{\left(\upbeta {\mathrm{X}}_{\mathrm{ik}}+{\upvarepsilon}_{\mathrm{ik}}+{\mathrm{U}}_{\mathrm{k}}\right)}$.
  7. Generate survival times *T*_*ik*_^*c*^ and *T*_*ik*_^*m*^. These survival distributions correspond to the respective hazard functions.
  8. Generate the censoring indicator *C*_*ik*_, The total observed trial data is then {*Y*_*ik*_, *C*_*ik*_, *Z*_*ik*_, *X*_*ik*_}, a set of censored survival data, treatment allocations and treatments received.
  9. Fit a Cox proportional hazards model to the data with respect to the four analysis methods ITT, PP, 2SRI and 2SPS, to produce an estimate ${\widehat{\upbeta}}_{\mathrm{j}}$ of the intervention effect *β*, which is the log of the hazard ratio, and record the *p*-value, *p*_*j*_.

When j = 1000, calculate the mean $\overline{\upbeta}={\displaystyle {\sum}_{\mathrm{j}}{\widehat{\upbeta}}_{\mathrm{j}}/1000}$, the percentage bias $\left(\overline{\beta}-\beta \right)\kern0.1em /\kern0.1em \beta$ and the statistical power ∑_j_I(p_j_ < 0.05)/1000. Also, calculate a parametrically bootstrapped standard error of the individual estimate $s.\;e.\;\left(\widehat{\beta}\right)=s.\;d.\;\left({\widehat{\upbeta}}_{\mathrm{j}}\right)$, the standard error of the mean $s.\;e.\;\left(\overline{\beta}\right)=s.\;d.\left({\widehat{\upbeta}}_{\mathrm{j}}\right)/1000$, and a confidence interval for the percentage bias $\mathrm{C}\mathrm{I}=\Big[100*\left(\left(\overline{\upbeta}-1.96*\kern0.5em s.\;e.\;\left(\overline{\beta}\right)\right)-\beta \right)/\beta, 100*\left(\left(\overline{\upbeta}+1.96*s.\;e.\;\left(\overline{\beta}\right)\right)-\beta \right)/\beta$.

Different scenarios are created by varying the following variables. The intra-cluster coefficient (ICC) takes values 0.025 and 0.05, simulated by (σ_ε_^2^, σ_u_^2^) = (0.6, 0.2) and (0.57, 0.27) respectively. Average patient and clinician refusal probabilities *p* and *q* take values 0.1, 0.2 and 0.3. The correlation between refusal probability and risk, *ρ*_*p*_, takes values zero, low, medium and high, simulated by having lower limits and upper limits for individual refusal probabilities as $\left(LL,UL\right)\in \left\{\left(p,p\right),\left(\frac{2p}{3},\frac{4p}{3}\right),\left(\frac{p}{3},\frac{5p}{3}\right),\left(0,2p\right)\right\}$. The correlation between clinician refusal and risk takes the same set of values. The reason for this structure is to give control over the correlation between individual risk and refusal probabilities. The treatment effect is fixed at *β* = − 0.32, which equates to on average a 25 % reduction in 10 year risk of CVD. 1000 independent sets of independent trial data are generated for each scenario.

Sample sizes are calculated at a fixed ratio of 4:1 control to study intervention, the type 1 error is 0.05 and required power is 0.8. Sample sizes are calculated through simulation [33] as sample size formulas for informatively censored clustered survival data are not common. Trials characteristics (effect size, refusal rate, baseline risk) are assumed to be known. Trial data is simulated using the above process and analysed using ITT. For each combination of refusal rates, the smallest N (that is a multiple of J = 620) such that the proportion of *p*-values < 0.05 is 80 % is chosen as the required sample size in that scenario. There are then two recruitment methods which alter the required sample size. Recruitment method 1 calculates the sample size assuming no refusal. Recruitment method 2 factors in the refusal rate in the sample size calculation (assuming refusal to be non-informative and independent of individual risks). All simulation scenarios are run using both recruitment methods. The power realised varies from 0.8 as we use the smallest number of clusters that achieve at least a power of 0.8, in recruitment method 2 this changes depending on the refusal rate.

The outcome of interest is the time until a CVD event so cox proportional hazards models are fitted to produce estimates for the intervention effect. To account for the clustering of the data, three types of Cox proportional hazard model are fitted: marginal, lognormal frailty, and gamma frailty models [34, 35]. The lognormal model is correctly specified because the generated random effects (frailties) are normally distributed (Table 1), whereas the gamma frailty model is miss specified. The output from the robust marginal model has a different interpretation to the frailty models in that the hazard ratio returned is between any two randomly selected patients from the population, as opposed to the hazard ratio of any two people randomly selected from the same cluster [35]. Clustering is not taken into account in the first stage of the IV model as the inclusion of residuals in the second stage model (2SRI) is expected to take account of variation in refusal rates between clusters.

## Results

**Fig. 1:**
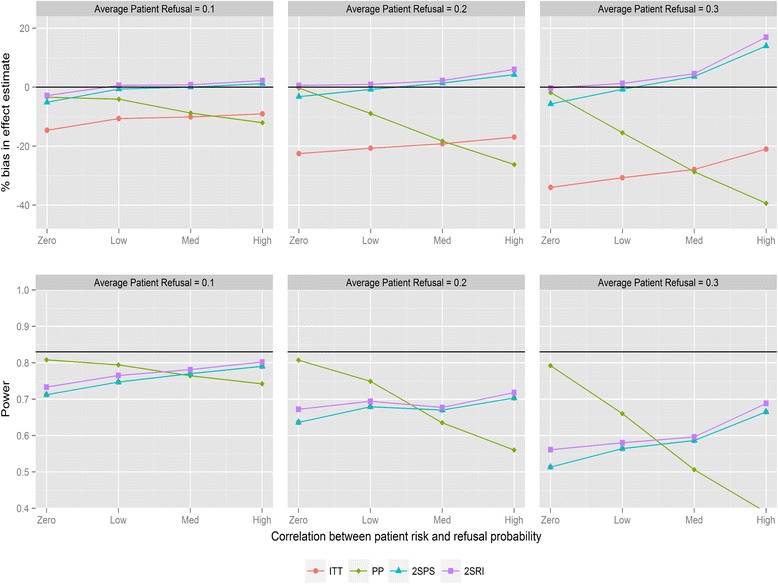
Percentage bias and power of the four analysis methods for varying levels of patient refusal and correlation between individual patient refusal probabilities and risk. Clinician refusal = 0, correlation is negative, recruitment method 1 is used and ICC =0.025. The black line in the power graph represents the expected power in the trial. A lognormal frailty model is fitted to the data

**Fig. 2:**
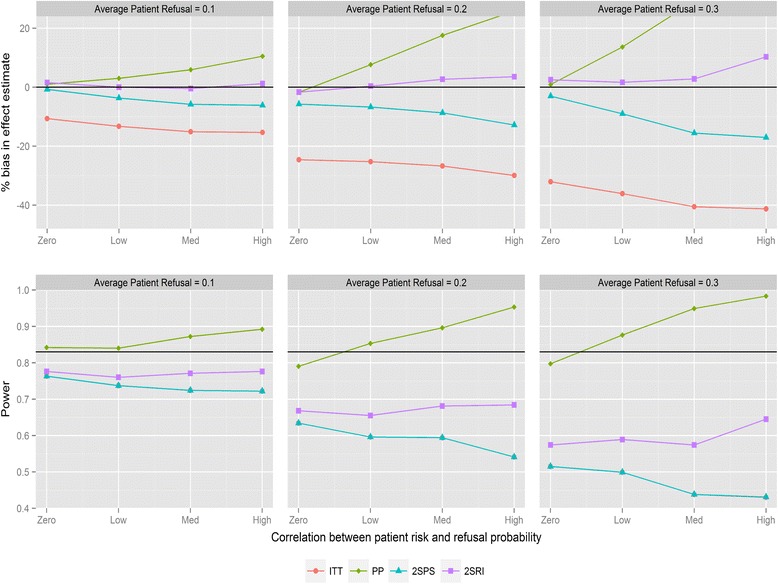
Percentage bias and power of the four analysis methods for varying levels of patient refusal and correlation between individual patient refusal probabilities and risk. Clinician refusal = 0, correlation is positive, recruitment method 1 is used and ICC =0.025. The black line in the power graph represents the expected power in the trial. A lognormal frailty model is fitted to the data

Figure 1 shows the magnitude of bias and loss of statistical power for the four analysis methods for varying average refusal rates and negative correlation between refusal and individual risk (for recruitment method 1). As expected, ITT underestimates the treatment effect. Refusal probabilities as small as 0.1 lead to bias between 9 and 16 %, refusal of 0.2 between 18 and 30 %, and refusal of 0.3 between 21 and 42 %, depending on the direction of the correlation. PP provides the most biased effect estimates when correlations are large with substantial reductions in statistical power. The two IV analyses provide similar results and substantially less biased effect estimates compared with ITT. The bias with both IV methods is below 6 % in all scenarios except when refusal is high with negative correlation, in which case 2SPS and 2SRI overestimate the effect of the study intervention by 13 and 17 % respectively. All methods have reduced statistical power with increasing refusals. ITT has the same statistical power as the 2SPS method in nearly all scenarios (i.e., the lines in Fig. 1 overlapping). For positive correlation (Fig. 2) 2SRI provides effect estimates with the lowest level of bias compared to the three other analysis methods, although it is associated with a statistical power between 0.8 and 0.56 rather than the 0.83 obtained from the sample size calculation. The trends in bias and power for ITT and 2SPS change direction with the correlation causing an increase in bias and a drop in power.

**Fig. 3:**
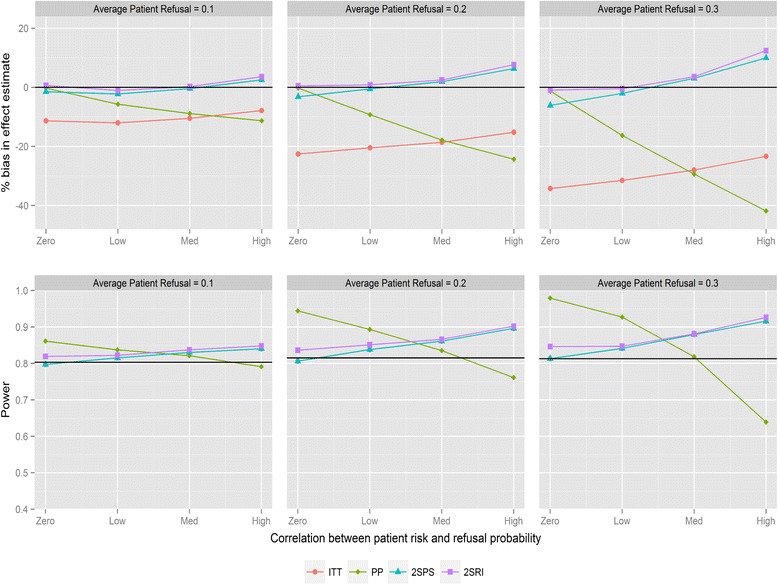
Percentage bias and power of the four analysis methods for varying levels of patient refusal and correlation between individual patient refusal probabilities and risk. Clinician refusal = 0, correlation is negative, recruitment method 2 is used and ICC =0.025. The black line in the power graph represents the expected power in the trial. A lognormal frailty model is fitted to the data

**Fig. 4:**
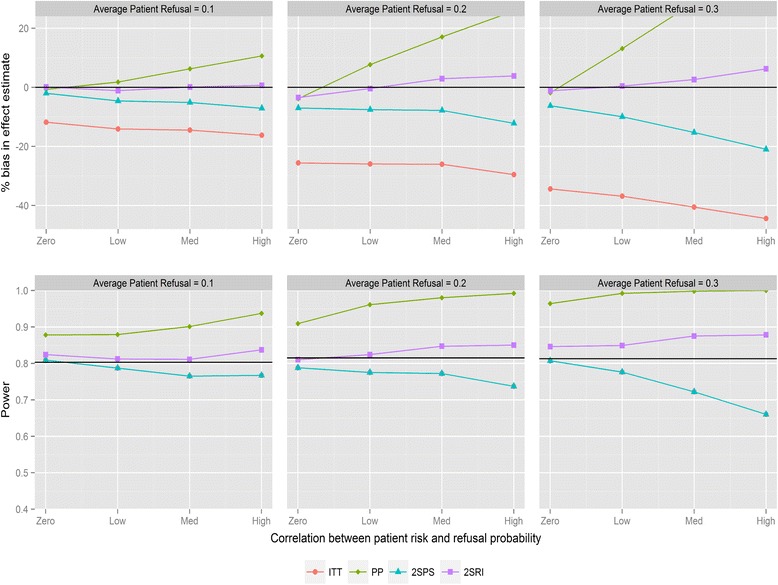
Percentage bias and power of the four analysis methods for varying levels of patient refusal and correlation between individual patient refusal probabilities and risk. Clinician refusal = 0, correlation is positive, recruitment method 2 is used and ICC =0.025. The black line in the power graph represents the expected power in the trial. A lognormal frailty model is fitted to the data

Figure 3 shows the results for recruitment method 2 and negative correlation between refusal and individual risk. There is no difference from recruitment method 1 with respect to bias or trends in statistical power; however the overall power of the trial stays consistent as refusal probabilities are changed. The only visible effect of changing refusal probabilities is to strengthen the effect of changing correlation. Importantly, the power of ITT and IV methods tends not to drop below the desired level. With positive correlations and recruitment method 2 (Fig. 4) there is a similar pattern when comparing to recruitment method 1. 2SRI provides the least biased estimates with the statistical power ranging between 0.81 and 0.88, depending on refusal. 2SPS and ITT yield more biased estimates with increasing reductions in statistical power as the rate of refusal increased.

**Fig. 5:**
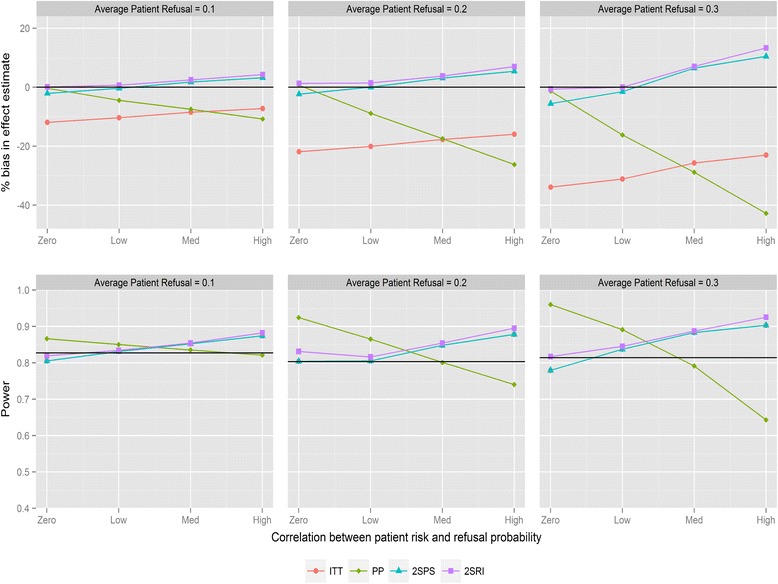
Percentage bias and power of the four analysis methods for varying levels of patient refusal and correlation between individual patient refusal probabilities and risk. Clinician refusal = 0, correlation is negative, recruitment method 2 is used and ICC =0.025. The black line in the power graph represents the expected power in the trial. A robust marginal frailty model is fitted to the data

**Fig. 6:**
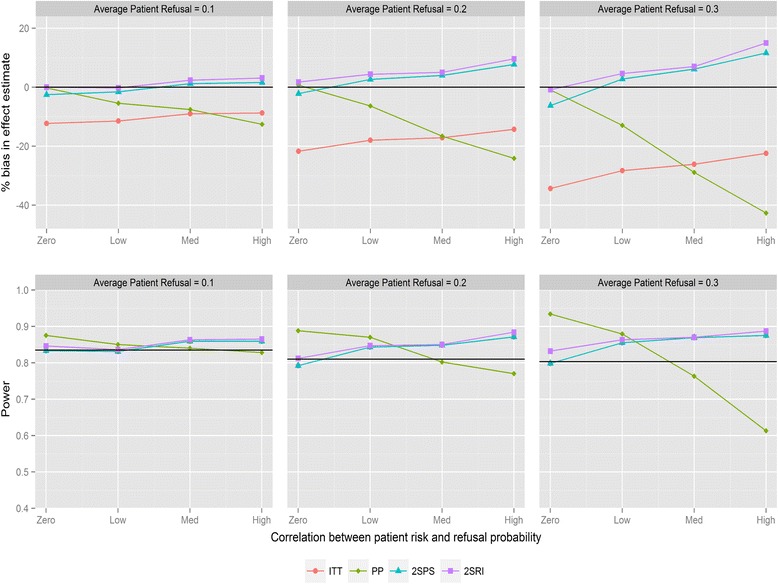
Percentage bias and power of the four analysis methods for varying levels of patient refusal and correlation between individual patient refusal probabilities and risk. Clinician refusal = 0, correlation is negative, recruitment method 2 is used and ICC =0.025. The black line in the power graph represents the expected power in the trial. A robust gamma frailty model is fitted to the data

Sensitivity analyses are conducted evaluating the effects of miss-specification of the statistical models. Figure 5 and Fig. 6 show the estimates and statistical power with a robust marginal and gamma frailty model fitted to the data respectively (for recruitment method 2 and negative correlations). The miss-specified models perform slightly worse than the correctly specified models in terms of percentage bias while the statistical power is slightly higher. If there was a greater variation between clusters, you would expect a lack of collapsibility to cause a difference between the marginal and frailty estimates [36]. This should be considered along with what output is desired (conditional/marginal) if running a cluster cmRCT. Results for positive correlations also did not vary greatly with model miss-specifications (data not shown).

In Appendix A a look into the accuracy of the bias estimates are provided and results from scenarios including changes in the ICC and when clinician refusal > 0.

## Discussion

This study has shown that refusal of a novel intervention in a cluster cmRCT design can lead to bias and reductions in power. The ITT estimates have a high bias, which increases with increasing refusal and is affected by correlations between refusal and the risk of outcome of interest. IV analyses using the 2SRI method substantially reduce the bias but yield small overestimates (generally < 6 %) of the treatment effect when refusal rates are high and correlation strong. The 2SPS estimates for IV are highly affected by the correlation structure and produce very biased estimates with positive correlation. Recruitment method 1 causes a loss in statistical power irrelevant of the analysis method used. On the other hand, cluster cmRCT are correctly powered or overpowered for recruitment method 2.

This study found, as expected, that refusals can lead to a large underestimate of the treatment effect when ITT is used (also known as dilution bias) [37]. Despite this, the published protocols of cmRCT either propose ITT for the primary analysis [9, 11, 12] or methods have not been stated yet [10, 13–16]. This follows the recommendation to use ITT for the analysis of pragmatic trials [5, 24, 38]. The main argument for this is that treatment refusals do happen in actual clinical practice and ITT would thus evaluate the value of offering a treatment. However, as stated earlier, refusal rates may be different in trials (due to e.g. more complex consent and recruitment procedures) and the ITT effect will not reflect that in routine practice. In cmRCTs, randomisation precedes the recruitment and consent procedures, and thus may affect results more than traditional trials. The results presented here highlight the large biases associated with ITT when estimating the treatment effect. Our results indicate the importance of evaluating in a cmRCT whether refusal rates are higher than expected in actual clinical practice (is estimating the ITT effect valuable?) and whether this refusal may be related to the outcome as this may increase the bias in estimating the treatment effect. It has been reported that the risk of early discontinuation may be correlated with the risk of outcome of interest [39].

IV deals with refusal and non-compliance in RCTs to some extent [27, 40] and has been adopted in practice [41–43]. This study found that IV indeed minimised bias due to refusal. Of the published cmRCTs, only two trials RECTAL BOOST [9] and SPIN [11] propose to use IV, and it is as a secondary analysis or if refusal rates exceed a predefined limit. The two IV methods proposed are 2SPS and 2SRI. 2SPS is a simple extension of ITT and can be obtained by dividing the ITT estimate by the average treatment refusal. Our simulations show that 2SPS is generally more biased than 2SRI. We also show that 2SPS estimates are sensitive to the data structure, when the direction of correlation between refusal and risk changed so did the trend in effect estimates. In the scenarios where 2SPS is less biased than 2SRI (negative correlation), it is only by a small amount. This is in line with the findings by Terza et al. [25], who concluded that 2SRI is the more appropriate method for nonlinear models and we therefore recommend 2SRI should be used if an IV analysis is carried out. However the effect estimate of interest in a pragmatic trial (the ITT effect in routine practice) is likely to lie somewhere in between the ITT and IV estimates and contextual information is needed to assess this. For example, if refusal rates are particularly high (> 50 %) then the IV estimate is unlikely to represent the effect of this drug in routine practice. This is in line with the findings of van der Velden et al [44], who imply both ITT and IV analyses should be carried out to analyse the results of a cmRCT.

Recruitment method 1 causes a drop in statistical power when using ITT or IV methods. This drop in power can be explained by the dilution bias for ITT as the apparent treatment effect is smaller. Although the IV estimate is generally unbiased, it suffers the same drop in power due to larger standard errors resulting from using a two stage modelling technique. If refusal rates are factored into the sample size calculation (recruitment method 2) the statistical power improves as expected. There is a paucity of literature on the methods for powering a RCT in case of refusals when using IV; most literature deals with ITT [45–47]. Our study highlights the need to take into account treatment refusal into the power calculation. Methods, such as simulations, will need to be developed to adjust IV analyses (with 2SRI) for non-informative as well informative refusals.

A limitation of this study is that we have only considered a homogeneous treatment effect. The results of this study are thus not generalizable to a heterogeneous treatment effect. The results of [48] can be referred to when deciding on which method to use if a heterogeneous treatment effect is thought to be present. In the case of a heterogeneous treatment effect, under certain assumptions IV estimates the complier average causal effect (CACE) [28]. The results from IV analysis should be presented following procedures laid out by Swanson and Hernán [49]. A second limitation is that the simulations presented here are based on aggregate data which may not be relevant to certain populations. It is thus important that each cluster cmRCT assesses prior to the start of trial the likely scenarios for refusal, risk of outcomes and cluster sizes and then estimate the potential impact on statistical power and level of bias. This would then guide the feasibility of the cluster cmRCT and the required sample size.

## Conclusions

In conclusion, cluster cmRCT can be an efficient design for conducting pragmatic trials, however there are still many questions to answer: What is the impact on bias and power when multiple trials are conducted within a cohort? What would be the influence of effect modification? What will happen if there is an overlap is secondary endpoints? The question addressed in this paper is how to deal with differential refusal in the intervention arm. If the refusal in a cmRCT is similar to that in routine clinical practice, then an ITT analysis will provide a valid estimate of the ITT effect in routine practice. If refusal differs from that in routine clinical practice, an IV analysis may provide a more accurate estimate. More research is needed on how refusal rates of experimental interventions correlate with refusal rates in routine practice to help answer the question of which analysis more relevant. For now, we recommend running both analyses and providing context about the intervention. Refusals can also adversely affect the statistical power of a cluster cmRCT and should be incorporated into the power calculation. An incorrect estimate of the refusal rate can lead to the recruitment of more patients than necessary or an underpowered trial. Therefore we recommend updating the required sample size during the trial as more information about the refusal rate is gained. Finally, it is important to note that the results, recommendations and discussion raised in this paper are very much applicable to the standard cmRCT design.

## Appendix A

In scenarios where clinician refusal is bigger than 0 the trends seen in Figs. 1, 2, 3 and 4 carry over to the changes in clinician refusal and correlation. The main result to report is that 2SRI performs similarly in all scenarios. In fact the bias only reaches over 6 % in one scenario where clinician refusal and correlation are 0.1 and zero (Fig. 1). This shows the 2SRI method is only slightly biased even as refusal rates and correlation strengths are quite large. In the same scenarios when the ICC = 0.05 the required sample sizes for each recruitment method changed, however the biases and power in each scenario were no different. The standard error of individual IV estimates ($s.\;e.\;\left(\widehat{\beta}\right)$), which have not been reported, are always larger than that of ITT and PP. In recruitment method 1 the standard error of the IV methods increases to around 0.15, almost half the magnitude of *β*. In recruitment method 2 the size of the standard errors did not change from around 0.11 (reflected by the consistent power). The 95 % confidence intervals around the percentage bias calculated is always between ∓ 2 – 3 % of the bias shown.

## Abbreviations

2SPS: Two stage predictor substitution
2SRI: Two stage residual inclusion
CACE: Complier average causal effect
cmRCT: Cohort multiple randomised controlled trial
CVD: Cardiovascular disease
HSCIC: Health and social care information centre
ITT: Intention to treat
IV: Instrumental variable
NICE: National institute for health and care excellence
PP: Per protocol
RCT: Randomised controlled trial

## References

[1] Vickers AJ (2014). Clinical trials in crisis: Four simple methodologic fixes. Clin Trials.

[2] Zwarenstein M, Oxman A (2006). Why are so few randomized trials useful, and what can we do about it?. J Clin Epidemiol.

[3] Zwarenstein M, Treweek S (2009). What kind of randomised trials do patients and clinicians need?. Evid Based Med.

[4] Luce BR, Kramer JM, Goodman SN, Connor JT, Tunis S, Whicher D, Schwartz JS (2009). Medicine and Public Issues Annals of Internal Medicine Rethinking Randomized Clinical Trials for Comparative Effectiveness Research: The Need for Transformational Change. Ann Intern Med..

[5] Thorpe KE, Zwarenstein M, Oxman AD, Treweek S, Furberg CD, Altman DG, Tunis S, Bergel E, Harvey I, Magid DJ, Chalkidou K (2009). A pragmatic-explanatory continuum indicator summary (PRECIS): a tool to help trial designers. J Clin Epidemiol.

[6] van Staa T-P, Goldacre B, Gulliford M, Cassell J, Pirmohamed M, Taweel A, Dalaney B, Smeeth L (2012). Pragmatic randomised trials using routine electronic health records: putting them to the test. BMJ.

[7] Purgato M, Barbui C, Stroup S, Adams C (2014). Pragmatic design in randomized controlled trials. Psychol Med.

[8] Relton C, Torgerson D, O’Cathain A, Nicholl J (2010). Rethinking pragmatic randomised controlled trials: introducing the ‘cohort multiple randomised controlled trial’ design. BMJ.

[9] Burbach JM, Verkooijen HM, Intven M, Kleijnen J-PJ, Bosman ME, Raaymakers BW, van Grevenstein WM, Koopman M, Seravalli E, van Asselen B, Reerink O (2015). RandomizEd controlled trial for pre-operAtive dose-escaLation BOOST in locally advanced rectal cancer (RECTAL BOOST study): study protocol for a randomized controlled trial. Trials.

[10] Mitchell N, Hewitt C, Adamson J, Parrott S, Torgerson D, Ekers D, Holmes J, Lester H, McMillan D, Richards D, Spilsbury K, Godfrey C, Gilbody S (2011). A randomised evaluation of CollAborative care and active surveillance for Screen-Positive EldeRs with sub-threshold depression (CASPER): study protocol for a randomized controlled trial. Trials.

[11] Kwakkenbos L, Jewett LR, Baron M, Bartlett SJ, Furst D, Gottesman K, Khanna D, Malcarne VL, Mayes MD, Mouthon L, Poiraudeau S, Sauve M, Nielson WR, Poole JL, Assassi S, Boutron I, Ells C, van den Ende CH, Hudson M, Impens A, Körner A, Leite C, Costa Maia A, Mendelson C, Pope J, Steele RJ, Suarez-Almazor ME, Ahmed S, Coronado-Montoya S, Delisle VC, Gholizadeh S, Jang Y, Levis B, Milette K, Mills SD, Razykov I, Fox RS, Thombs BD (2013). The Scleroderma Patient-centered Intervention Network (SPIN) Cohort: protocol for a cohort multiple randomised controlled trial (cmRCT) design to support trials of psychosocial and rehabilitation interventions in a rare disease context. BMJ Open.

[12] Uher R, Cumby J, Mackenzie LE, Morash-conway J, Glover JM, Aylott A, Propper L, Abidi S, Bagnell A, Pavlova B, Hajek T, Lovas D, Pajer K, Gardner W, Levy A, Alda M (2014). A familial risk enriched cohort as a platform for testing early interventions to prevent severe mental illness. BMC Psychiatry.

[13] Young-Afat HMVDA, van Gils CH, van den Bongard HJ, van Vulpen M (2014). Introducing the new ‘cohort multiple Randomised Controlled Trial’ design for evaluation of interventions for breast cancer: The UMBRELLA study. Eur J Cancer.

[14] R. Uher, “Skills for Wellness - Full Text View - ClinicalTrials.gov,” 2013. [Online]. Available: https://clinicaltrials.gov/show/NCT01980147. [Accessed: 29-Oct-2015].

[15] Bower P. ISRCTN - ISRCTN12286422: CLASSIC Proactive Telephone Coaching and Tailored Support (PROTECTS). 2014.

[16] M. van Vulpen, “Randomized Trial Comparing Conventional Radiotherapy With Stereotactic Radiotherapy in Patients With Spinal Metastases - VERTICAL Study - Full Text View - ClinicalTrials.gov,” 2015. [Online]. Available: https://www.clinicaltrials.gov/ct2/show/NCT02364115. [Accessed: 29-Oct-2015].

[17] van Staa TP, Dyson L, McCann G, Padmanabhan S, Belatri R, Goldacre B, Cassell J, Pirmohamed M, Torgerson D, Ronaldson S, Adamson J, Taweel A, Delaney B, Mahmood S, Baracaia S, Round T, Fox R, Hunter T, Gulliford M, Smeeth L (2014). The opportunities and challenges of pragmatic point-of-care randomised trials using routinely collected electronic records: Evaluations of two exemplar trials. Health Technol Assess (Rockv).

[18] Angrist JD, Imbens GW, Rubin DB, Association S, Jun N (1996). Identification of Causal Effects Using Instrumental Variables. J Am Stat Assoc.

[19] Greenland S (2000). An introduction to instrumental variables for epidemiologists. Int J Epidemiol.

[20] Frangakis BCE, Rubin DB (1999). Addressing complications of intention-to-treat analysis in the combined presence of all-or-none treatment-noncompliance and subsequent missing outcomes. Biometrika.

[21] Dunn G, Maracy M, Tomenson B (2005). Estimating treatment effects from randomized clinical trials with noncompliance and loss to follow-up: the role of instrumental variable methods. Stat Methods Med Res.

[22] Wu J, Zhu S, Yao GL, Mohammed MA, Marshall T (2013). Patient Factors Influencing the Prescribing of Lipid Lowering Drugs for Primary Prevention of Cardiovascular Disease in UK General Practice: A National Retrospective Cohort Study. PLoS One.

[23] Roland M, Torgerson DJ (1998). Understanding controlled trials: What are pragmatic trials?. BMJ.

[24] Armijo-Olivo S, Warren S, Magee D (2009). Intention to treat analysis, compliance, drop-outs and how to deal with missing data in clinical research: a review. Phys Ther Rev.

[25] Terza JV, Basu A, Rathouz PJ (2008). Two-stage residual inclusion estimation: Addressing endogeneity in health econometric modeling. J Health Econ.

[26] Efron BYB (1981). Nonparametric estimates of standard error: The jackknife, the bootstrap and other methods. Biometrika.

[27] Groenwold RHH, Uddin MJ, Roes KCB, De Boer A, Martin E, Gatto NM, Klungel OH (2014). Instrumental variable analysis in randomized trials with non- compliance and observational pharmacoepidemiologic studies. OA Epidemiology.

[28] Hernán MA, Robins JM (2006). Instruments for causal inference: an epidemiologist’s dream?. Epidemiology.

[29] HSCIC, “Numbers of Patients Registered at a GP Practice - April 2015,” 2015. [Online]. Available: http://www.hscic.gov.uk/searchcatalogue?productid=17788&topics=1%2fPrimary+care+services%2fGeneral+practice&sort=Most+recent&size=10&page=2#top. [Accessed: 30-Oct-2015].

[30] Townsend N, Williams J, Bhatnagar P, Wickramasinghe K, Rayner M (2014). Cardiovascular disease statistics, 2014. British Heart Foundation: London. 10.1136/heartjnl-2015-307516 PMC4515998 26041770

[31] Eldridge SM, Ashby D, Kerry S (2006). Sample size for cluster randomized trials: Effect of coefficient of variation of cluster size and analysis method. Int J Epidemiol.

[32] Kerry SM, Martin Bland J (2001). Unequal cluster sizes for trials in English and Welsh general practice: Implications for sample size calculations. Stat Med.

[33] Arnold BF, Hogan DR, Colford JMJ, Hubbard AE (2011). Simulation methods to estimate design power: an overview for applied research. BMC Med Res Methodol.

[34] O’Quigley J, Stare J (2002). Proportional hazards models with frailties and random effects. Stat Med.

[35] Glidden DV, Vittinghoff E (2004). Modelling clustered survival data from multicentre clinical trials. Stat Med.

[36] Greenland S, Robins J, Pearl J (1999). Confounding and collapsibility in causal inference. Stat Sci.

[37] Adamson J, Cockayne S, Puffer S, Torgerson DJ (2006). Review of randomised trials using the post-randomised consent (Zelen’s) design. Contemp Clin Trials.

[38] Hollis S, Campbell F (1999). What is meant by intention to treat analysis? Survey of published randomised controlled trials. BMJ.

[39] Snapinn SM, Jiang Q, Iglewicz B (2004). Informative noncompliance in endpoint trials. Curr Control Trials Cardiovasc Med.

[40] Ye C, Beyene J, Browne G, Thabane L (2014). Estimating treatment effects in randomised controlled trials with non-compliance: a simulation study. BMJ Open.

[41] Goldsmith LP, Lewis SW, Dunn G, Bentall RP (2015). Psychological treatments for early psychosis can be beneficial or harmful, depending on the therapeutic alliance: an instrumental variable analysis. Psychol Med.

[42] Jago R, Edwards MJ, Sebire SJ, Tomkinson K, Bird EL, Banfield K, May T, Kesten JM, Cooper AR, Powell JE, Blair PS (2015). Effect and cost of an after-school dance programme on the physical activity of 11–12 year old girls: The Bristol Girls Dance Project, a school-based cluster randomised controlled trial. Int J Behav Nutr Phys Act.

[43] Halpern SD, French B, Small DS, Saulsgiver K, Harhay MO, Audrain-McGovern J, Loewenstein G, Brennan TA, Asch DA, Volpp KG (2015). Randomized Trial of Four Financial-Incentive Programs for Smoking Cessation. N Engl J Med.

[44] van der Velden JM, Verkooijen HM, Young-Afat DA, Burbach JPM, van Vulpen M, Relton C, van Gils CH, May AM, Groenwold RHH. The cohort multiple randomized controlled trial design: a valid and efficient alternative to pragmatic trials?. Int J Epidemiol. 2016. doi:10.1093/ije/dyw050. 10.1093/ije/dyw050 27118559

[45] Lakatos E (1986). Sample size determination in clinical trials with time-dependent rates of losses and noncompliance. Control Clin Trials.

[46] Sato T (2000). Sample size calculations with compliance information. Stat Med.

[47] Jiang Q, Snapinn S, Iglewicz B (2004). Calculation of sample size in survival trials: the impact of informative noncompliance. Biometrics.

[48] Wan F, Small D, Bekelman JE, Mitra N (2015). Bias in estimating the causal hazard ratio when using two-stage instrumental variable methods. Stat Med.

[49] Swanson SA, Hernán MA (2013). Commentary. Epidemiology.

